# Smart Glasses-Based Personnel Proximity Warning System for Improving Pedestrian Safety in Construction and Mining Sites

**DOI:** 10.3390/ijerph17041422

**Published:** 2020-02-22

**Authors:** Jieun Baek, Yosoon Choi

**Affiliations:** Department of Energy Resources Engineering, Pukyong National University, Busan 48513, Korea; bje0511@gmail.com

**Keywords:** smart glass, Bluetooth beacon, pedestrian safety, personnel proximity warning system

## Abstract

A smart glasses-based wearable personnel proximity warning system (PWS) was developed for pedestrian safety in construction and mining sites. The smart glasses receive signals transmitted by Bluetooth beacons attached to heavy equipment or vehicles, with the proximity determined by the signal strength. A visual alert is displayed to the wearer when in close proximity. The media access control address of the Bluetooth beacon provides information on the approaching equipment or vehicle, which is displayed to the wearer so that they can respond appropriately. There was a detection distance of at least 10 m regardless of the direction the pedestrian was looking and the alert was successful in all 40 trials at ≥10 meters. The subjective workload was evaluated using the NASA task load index on ten subjects, either without a personal PWS, with a smartphone-based PWS, or with the smart glasses-based PWS. The mental, temporal, and physical stresses were lowest when using the smart glasses-based PWS. Smart glasses-based PWSs can improve work efficiency by freeing both hands of the pedestrians, and various functions can be supported through application development. Therefore, they are particularly useful for pedestrian safety in construction and mining sites.

## 1. Introduction

Collisions between heavy construction equipment and workers occur frequently in construction and mining sites around the world. According to the U.S. Bureau of Labor Statistics, 27% of the 1083 casualties in construction and mining in the USA in 2017 were related to collisions with equipment [1]. Eurostat reported 31 fatalities related to collisions with equipment at construction and mining sites in Europe between 2014 and 2018 [2]. In Korea, there were 121 fatalities due to collisions with the power machinery used at mining and construction sites in 2018 [3]. To prevent such collisions, proximity warning systems (PWSs) should be implemented at construction and mining sites [4].

PWSs provide proximity alarms to warn workers about approaching equipment or pedestrians [5]. They employ various wireless communication technologies, including electromagnetic fields (e.g., HazardAvert, Strata Worldwide, Sandy Springs, GA, USA [6]), global positioning system (GPS) (e.g., MineAlert, Modular Mining, Tucson, AZ, USA [7]), radio-frequency identification (RFID) (e.g., GE Collision Awareness System, GE Mining, Brisbane, Queensland, Australia [8]), and Wi-Fi (e.g., Proximity Detection & Collision Avoidance, Newtrax, Montreal, Quebec, Canada [9]). Electromagnetic PWSs comprise transmitters that generate electromagnetic signals using a wire-loop antenna and receivers that detect the signals when the two components are in close proximity [10,11]. GPS PWSs use GPS devices that are attached to equipment and workers to determine their locations, and provide alerts when collisions are likely [12]. They operate best in open-pit mine environments. RFID PWSs are the most commonly used commercially available PWSs, as they can recognize both near and far objects using ultra-high frequency and very-low frequency signals [13,14,15,16,17].

Recently, there has been development of PWSs using Bluetooth low-energy (BLE) technology. BLE is a short-range wireless communication technology of Bluetooth 4.0 version (Bluetooth SIG, Inc., Kirkland, WA, USA) released in 2010 [18]. It consumes about 90% less power than classic Bluetooth technology and enables fast data transmission [19,20]. Park et al. [21] developed a PWS that recognizes equipment and operators using BLE signals transmitted by Bluetooth beacons. Subsequently, they proposed a construction-safety monitoring system that recognizes the location of workers and equipment through BLE technology, detects the occurrence of hazards through building information model technology, and enables real-time communication through a cloud-based platform [22]. Baek and Choi [23] proposed a Bluetooth-beacon-based underground PWS that can be used in underground tunnels and mines. However, these PWSs provide drivers with visual or auditory proximity alerts via an in-cab screen such as a mounted tablet or smartphone. Therefore, it is difficult for these PWSs to provide proximity warnings to pedestrians.

To overcome this limitation, wearable PWSs have been developed. Wearable PWSs should be small and light enough to be attached to a helmet or safety vest or be carried in a pocket. Jobes et al. [24] developed a personal alarm device that uses the triangulation of magnetic fields to estimate the wearer’s distance from continuous mining machines, and provides audible alerts to the wearer when in close proximity of the machine. Park et al. [25] proposed a BLE proximity sensing and alert system that uses a smartphone to receive BLE signals and provides proximity alerts based on the signal strength. Although several wearable PWSs for pedestrians have been developed, no personal PWSs providing workers with visual proximity alarms through smart glasses have been announced. In addition, no studies have been conducted on the subjective workload felt by pedestrians when wearing smart glasses-based personal PWSs.

Smart glasses are optical head mounted displays that project a virtual image that is visible to the wearer on top of the real-world view [26,27]. The development of smart glasses began when the concept of augmented reality was defined by Caudell and Mizell [28] in 1992. In 2013, Google released “Google Glass,” which created huge interest in smart glasses. Since then, Sony, Microsoft, and Epson have also announced smart glasses [29]. Smart glasses are used in various fields including healthcare [30,31,32], training [33,34], logistics [35,36], and tourism [37,38], with many commercially available products. Detailed information on commercially available smart glasses have been reported by Syberfeldt et al. [39], as shown in Table 1.

In this study, we developed a smart glasses-based personal PWS for pedestrian safety in construction and mining sites. By attaching Bluetooth beacons to heavy equipment or vehicles, which transmit signals that are received by the smart glasses, the proximity of the vehicle can be determined based on the signal strength. The pedestrian receives a visual alert when in close proximity to the vehicle. We tested the developed personal PWS at real mining sites to determine its performance, and analyzed the subjective workload felt by pedestrians using the NASA task load index (NASA-TLX) [40].

## 2. Methods

### 2.1. Development of Personal PWS Using Smart Glasses

**Figure 1:**
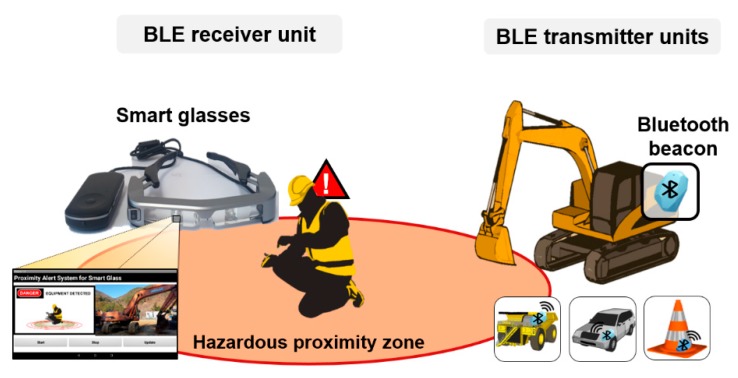
Conceptual view of the personal proximity warning system (PWS) comprising a Bluetooth low energy (BLE) receiver unit and BLE transmitter units.

The design of the personal PWS is summarized in Figure 1. Smart glasses worn by pedestrians receive BLE signals transmitted from Bluetooth beacons and provide visual alarms when in close proximity to a beacon. The Bluetooth beacons can be attached to heavy equipment, vehicles, or hazardous areas in construction or mining sites, and they continuously transmit BLE signals. Hence, as well as alerting the wearer to approaching heavy equipment or vehicles, the smart glasses can alert the wearer to the fact that they are approaching a hazardous area. The visual proximity alerts are received through the smart glasses while performing on-site work such as safety checks and maintenance, allowing workers to quickly detect and respond to dangerous situations.

BLE technology can provide point-to-point, broadcast, or mesh wireless communication [41]. In point-to-point communication, data are exchanged by connecting the “master” device (which transmits data) and the “slave” device (which receives data) in a 1:1 configuration. In broadcast communication, several observers are able to receive the ID information transmitted periodically by a single broadcaster. In mesh communication, numerous master and slave devices are connected to exchange data.

Bluetooth beacons periodically transmit BLE signals with information on the universally unique identifier of the beacon, the media access control (MAC) address, and more. Therefore, the Bluetooth beacons act as broadcasters, while computers, smartphones, and smart glasses that can receive BLE signals are used as observers. The reception strength of the BLE signal can be quantified by the received signal strength index (RSSI), expressed as a number between −99 and −35 dBm. The propagation distance of the BLE signal can vary depending on the signal transmission strength and the signal propagation direction of the Bluetooth beacon. As the BLE signal transmission strength increases, the signal propagation distance increases. While the signal propagation direction can be set to be nondirectional to propagate the signal uniformly in all directions, this limits the propagation distance. If the signal is set to be directional, the BLE signal propagates relatively far ahead of the Bluetooth beacon. The changes in RSSI according to the BLE signal transmission strength and the propagation direction of the Bluetooth beacon have been analyzed previously [42].

**Figure 2:**
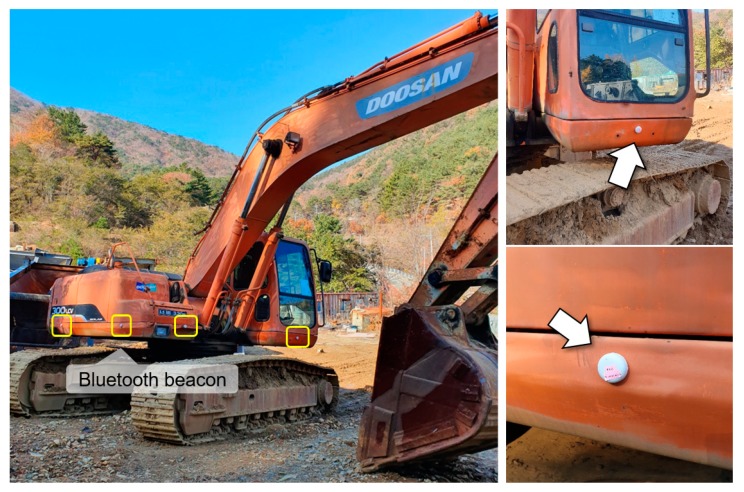
Installation of Bluetooth beacons on the excavator.

RECO beacons (Perples, Seoul, South Korea) were used as the BLE transmission unit. RECO beacons are certified by agencies in Korea, the USA, Europe, Japan, etc., and they are in line with global beacon standards. The main specifications of the RECO beacons are listed in Table 2. Figure 2 shows an example of heavy equipment at construction and mining sites with the RECO beacons attached. Two Bluetooth beacons were installed on the front, rear, and sides of the heavy equipment, giving it a total of eight beacons. The Bluetooth beacons were set to transmit directional signals, so that the signals would propagate farther. The signal transmission strength and period of the beacons were set to 4 dBm and 10 ms, respectively.

**Figure 3:**
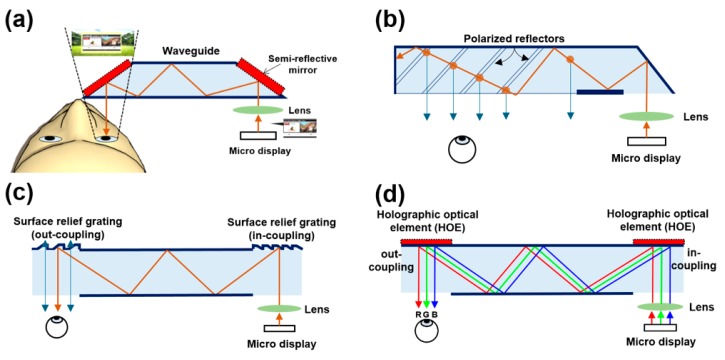
Mechanism of different waveguide-based optical see-through smart glasses displays: (**a**) reflective; (**b**) polarized; (**c**) diffractive; (**d**) holographic.

Smart glasses typically use optical see-through display technology to overlap the real-world and virtual environments. The virtual image is projected through the microdisplay into the lens and reflects light through a flat or a curved mirror to an optical combiner located in front of the human eye. The optical combiner superimposes the virtual image on top of the real-world view and delivers it to the user’s eyes. In this process, waveguides are used to guide the light to effectively propagate it to the human eye without any losses. Optical fibers or prisms are often used as waveguides. Waveguides can be classified into reflective-, polarized-, diffractive-, and holographic-type, depending on the arrangement and the reflector material (Figure 3). Reflective waveguides reflect and transmit virtual images using a semi-reflective mirror, while polarized waveguides reflect light through a series of polarized reflectors to deliver the image to the human eye. Diffractive waveguides reflect light through in- and out-coupling using a diffractive optical element with surface relief grating structures. Holographic waveguides are similar in principle to diffractive waveguides, where a holographic optical element is used to separate and transmit the red, green, and blue components of light. More information on waveguides can be found in Erdenebat et al. [43].

**Figure 4:**
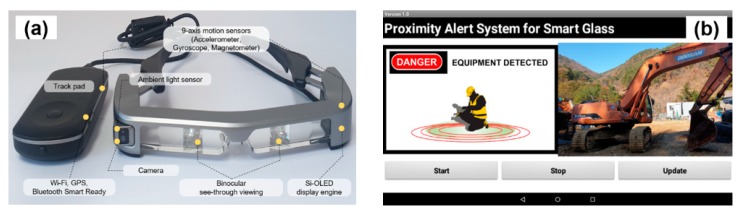
Overview of BLE receiver units: (**a**) appearance of Moverio BT-350 used for BLE receiver units; (**b**) user interface of the Android application providing proximity warning alarm.

In this study, Moverio BT-350 smart glasses (Epson, Suwa, Japan) were used as the wearable personal PWSs for pedestrians. These smart glasses consist of a headset and a controller, and provide a stereoscopic view to the user by a reflective waveguide method [44]. Figure 4a shows the appearance of the equipment, and Table 3 lists the specifications of the Moverio BT-350 model. This device uses the Android 5.1 operating system and is equipped with various instruments including GPS, a gyroscope, accelerometer, and geomagnetic sensors. In addition, Wi-Fi communication and Bluetooth 4.1 (Bluetooth Smart Ready Class 2) communication are possible. 

To control the personal PWS, we developed an application for the smart glasses for Android operating systems using the MIT App Inventor application [45]. Figure 4b shows the user interface of the developed application. The application visually alerts the pedestrian when the RSSI value of the BLE signal received by the smart glasses reaches a certain level. In addition, based on the MAC address of the Bluetooth beacon that transmits the BLE signal, information on the approaching device or the vehicle is also provided so that the pedestrians can respond effectively when in close proximity.

### 2.2. Performance Assessment of Smart Glasses-Based Personal PWS

**Figure 5:**
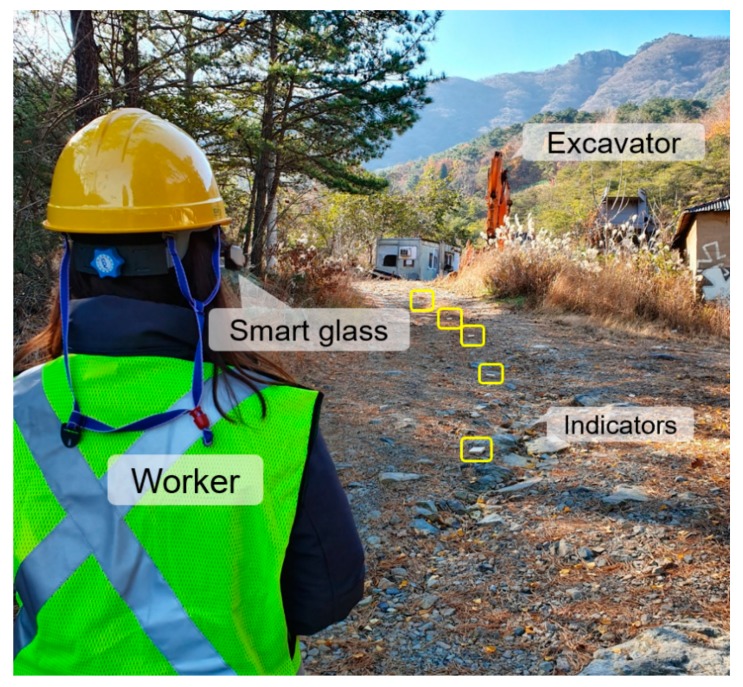
View of performance experiment of personal PWS at the Ilgwang mining site.

To evaluate the performance of the developed personal PWS, field experiments were conducted in the Ilgwang mining site (35°18’33” N, 129°13’35” E) located in Gijang-gun, Busan, Korea. As shown in Figure 2, a total of eight Bluetooth beacons were attached to the excavator, and pedestrians wore smart glasses installed with the personal PWS application. The controller of the smart glasses with a built-in BLE signal receiving antenna was positioned to face the front of the pedestrian. The excavator approached a pedestrian standing in the center of a mine hauling route from 40 m away at a speed of 10–20 km/h, as shown in Figure 5. We then measured the detection distance at which the personal PWS receiving the BLE signal began alerting the pedestrian. To determine the detection distance according to the direction of the receiver, the facing angles between the pedestrian and the excavator were set at 45° intervals from 0° to 315°, and the detection distance was measured five times for each of the eight angular conditions (a total of 40 measurements). The minimum safety distance between the pedestrian and excavator was set to 10 m, and it was determined whether proximity alerts were provided to pedestrians before the excavator approached within a safe distance.

### 2.3. Subjective Workload Assessment of Smart Glasses-Based Personal PWS

Workload is a quantitative measure of the amount of mental stress a person feels while performing tasks within a particular system [46]. Workload is affected by factors of psychological (focus on work, anxiety), physical (physical difficulties, difficulty in controlling machines), temporal (deadlines), and environmental (noise, relationships with colleagues) nature [47]. If there are many negative factors in the system, over- or under-load can occur, which can reduce work efficiency. Therefore, it is necessary to design and operate a system with minimal workload to improve the work efficiency.

Subjective workload evaluation can examine workload through a questionnaire. It is frequently used in human–machine system development [48]. Representative subjective workload evaluation methods include NASA-TLX [40], the subjective workload assessment technique [49], and the workload profile technique [50]. In this study, the subjective workload was evaluated using the NASA-TLX method to evaluate the psychological, physical, and temporal effects of pedestrians wearing smart glasses and using the personal PWS while working on a mining site. NASA-TLX is a multi-dimensional rating procedure that estimates the overall workload score based on a weighted average of six factors [51]: mental demand, physical demand, temporal demand, own performance, effort, and frustration level. These workload parameters are defined as follows:

- Mental demand: How much mental and cognitive skills were required to perform this task?
- Physical demand: How much physical ability was required to perform this task?
- Temporal demand: How much time pressure did you feel due to the rate or pace at which you performed multiple tasks?
- Own performance: How successfully do you think you have achieved the goal of this task?
- Effort: How much mental and physical efforts were required to achieve your work aims?
- Frustration level: How many uncomfortable feelings (stress, anger) did you feel while performing this task?

The degree to which the subject felt the six workload parameters is rated by the subject. All parameters except “own performance” (which is scored from Excellent to Poor) are scored from Low to High by a value between 0 and 100 (in increments of 5). Next, the weights of the six parameters are calculated using pairwise comparison, and the overall workload score is calculated by averaging the product of the scores and weight of each factor.

Subjective workload evaluation was performed on ten subjects aged 22–27 years (mean age 23.6 years) in the same location where the performance of personal PWS was evaluated. Most of the subjects (70%) said they had knowledge of smart glasses, and 20% said they had used smart glasses before. Three equivalent experiments were performed with the same experimental conditions to compare the effect on subjective workload, where the subjects (1) did not use a personal PWS, (2) used a smartphone-based personal PWS, and (3) used the smart glasses-based personal PWS. The smartphone-based personal PWS application was developed in this study and was installed on a Samsung Galaxy S9 (Samsung Electronics, Suwon, Korea) that supports Bluetooth 5.1.

In all experiments, the subject stood in the center of the hauling road to investigate the road maintenance status, while an excavator or general vehicle approached the subject. A proximity alert along with an image of the equipment was made visible to the subject using the smartphone or smart glasses. The subject was informed to move to an evacuation area outside the nearby transport road if the type of equipment was an excavator, while if the type of equipment was a normal vehicle, the work was paused and resumed once the vehicle had passed through. 

The ten subjects each performed all three experiments (1)–(3) in a random order, and after the experiment, the workload for the experiment was examined according to the NASA-TLX procedure.

## 3. Results

**Figure 6:**
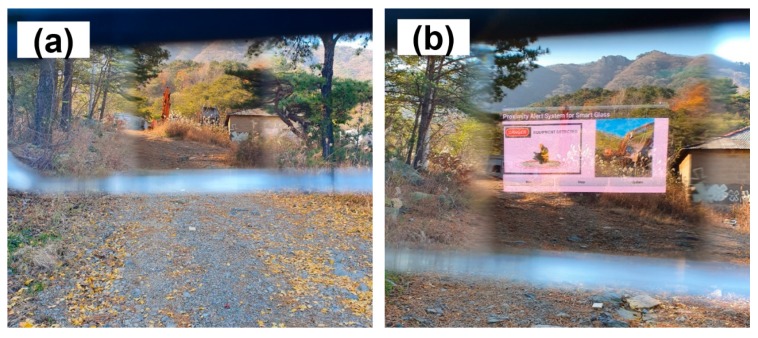
Results of performance experiment of personal PWS: (**a**) optical view of the smart glasses a safe distance away from the excavator; and (**b**) optical view of the smart glasses close to the excavator.

Figure 6a shows the smart glasses when no BLE signal was detected, and Figure 6b shows the visual alert screen when the BLE signal was recognized. Depending on the MAC address of the Bluetooth beacon that sent the BLE signal, a picture of the excavator already stored in the personal PWS application appears on the alert screen. Therefore, pedestrians wearing smart glasses can not only receive an alarm about the approaching heavy equipment, but they can also be warned about the type of heavy equipment.

**Figure 7:**
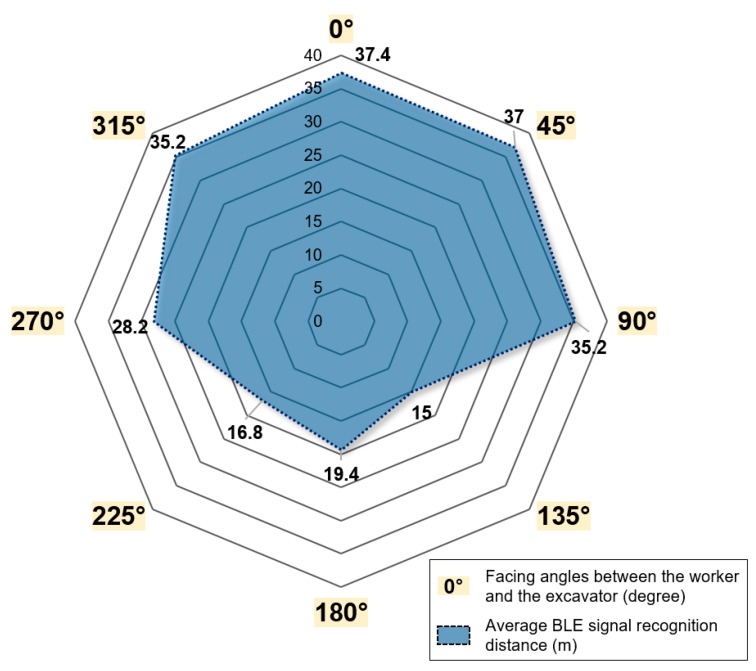
Average BLE signal recognition distance (m) of the smart glass according to facing angles between the worker and excavator.

Table 4 shows the main statistics of the detection distance measurement when the proximity alert is provided, depending on the facing angle between the pedestrian and the excavator, and Figure 7 is a radial representation of the average detection distance per angle. The average detection distance was more than 30 m when the facing angle was 0°, 45°, 90°, or 315°; more than 20 m when the angle was 270°; and 15–20 m when the angle was 135°, 180°, or 225°. The relatively short distance at the latter angles is likely to be because the BLE signal receiving antenna of the smart glasses and the Bluetooth beacon attached to the excavator are facing opposite directions. Nevertheless, the field experiments show that the developed personal PWS has a detection distance of at least 10 m regardless of the direction in which the pedestrian is looking. In all 40 experiments, alerts were always issued when the excavator approached the pedestrian within the minimum safety distance of 10 m.

**Figure 8:**
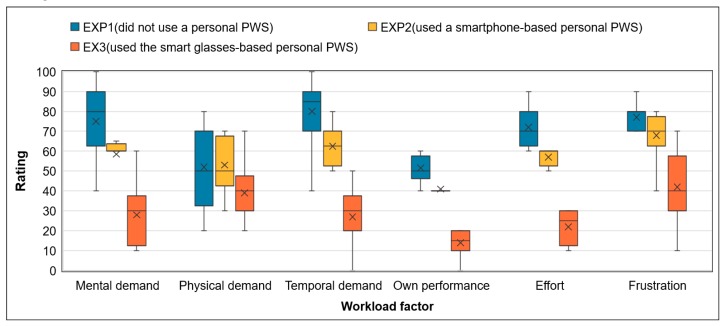
Results of rating six workload factors of NASA-TLX according to type of experiments.

Figure 8 shows the scores of the six workload parameters evaluated in three experiments on ten subjects. When the pedestrians did not use a personal PWS, the mental demands, temporal demands, and mental stress were high. This could be because the pedestrians needed to constantly check by eye for approaching vehicles and equipment while working. When the pedestrians used smartphone-based personal PWSs, they had to check their smartphone screen repeatedly to check for approaching excavators or vehicles, and because of increased eye movements, they felt negative emotions. In addition, since the pedestrians had to work with a smartphone in their hands, they did not have both hands free, so more effort was required to achieve their work task. When the pedestrians wore smart glasses-based personal PWSs, most of the workload factors, except for the frustration level, were low. The reason for the high level of frustration is that the subjects felt uncomfortable because they were not accustomed to wearing the smart glasses (the glasses sliding, wearing regular glasses under the smart glasses).

**Figure 9:**
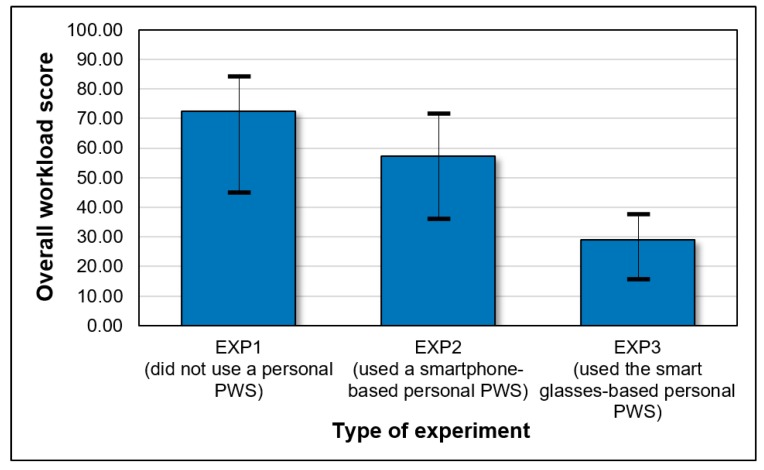
Overall workload score according to type of experiment.

Figure 9 shows the calculated overall workload scores from the three experiments. The overall workload score was about 72.4 for pedestrians not using a personal PWS, about 57.2 for those using a smartphone-based personal PWS, and about 28.9 for those wearing a smart glasses-based personal PWS. The mental, temporal, and psychological stresses of pedestrians were lower when wearing a smart glasses-based personal PWS than when not using a PWS or when using a smartphone-based PWS. Smart glasses free up both hands of pedestrians while effectively providing proximity alerts about equipment or vehicles, helping pedestrians to increase their work efficiency. However, some subjects felt uncomfortable with the fit of the smart glasses; hence, further study is necessary to improve the smart glasses wearability.

## 4. Discussion

### 4.1. Utilization of Smart Glasses-Based Personal PWS in Underground Tunnel

Collisions between equipment and pedestrians occur frequently in underground mines and tunnels. To verify that the smart glasses-based personal PWS could be used underground, a simple field experiment was additionally conducted in the Yeonhwa tunnel (35°12’55”N, 129°13’2”E) located in Gijang-gun, Busan, Korea.

Eight Bluetooth beacons were attached to a typical vehicle, with the Bluetooth beacons set to send directional signals. The smart glasses application was also updated by uploading the Bluetooth beacon’s MAC address and the photo of the vehicle to the database. Pedestrians waited inside the tunnel with smart glasses on, and the vehicle ran from the entrance to the end of the tunnel.

**Figure 10:**
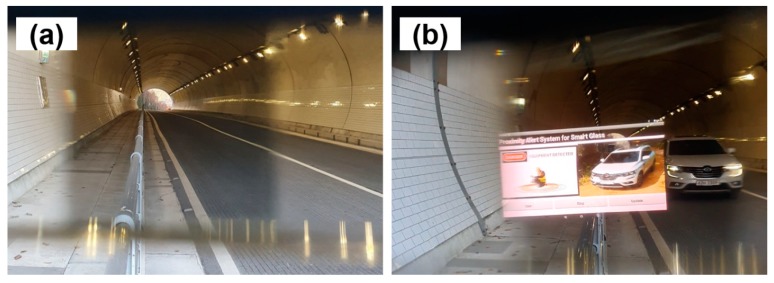
Examples of utilizing the personal PWS using smart glass in the underground tunnel: (**a**) no alert, and (**b**) visual proximity alerts.

Figure 10 shows the results of the field experiment inside the tunnel. When the vehicle was not inside the tunnel, no alert was displayed on the smart glasses (see Figure 10a). When the vehicle entered the tunnel and approached the worker, visual proximity alerts and a photo of the vehicle were visualized on the smart glasses (see Figure 10b). The experiment confirmed that the personal PWS could be used underground.

### 4.2. Advantages and Disadvantages of Smart Glasses-Based Personal PWS

There are two main problems with existing PWSs that provide workers with proximity alerts in visual (LED), audible, or vibration forms. First, PWSs are mainly intended for use on construction and mining sites, where vibration and noise are constantly generated by the operation of machines and equipment and frequent blasting work. This makes it difficult for the workers to easily recognize sound and vibration alerts. In addition, if earphones are worn to listen to the sound notifications, sound from the surrounding area is cut off, heightening the risk of various accidents. Second, these PWSs provide constant proximity alarms regardless of the type of equipment or vehicle approaching the operator. Instead, different types of proximity alarms should be provided for different types of equipment, to allow workers to quickly recognize the type of equipment approaching and quickly determine which action to take.

Personal PWSs using smart glasses can solve the problem of existing PWSs by providing workers with visual proximity alarms. Regardless of vibration and noise generated at construction and mining sites, workers can receive visual alerts through smart glasses, allowing the alert to be quickly identified, while hearing the surrounding sounds and vibrations. This helps pedestrian workers to respond quickly to dangerous situations. Smart glasses applications provide not only proximity alerts, but also information about the equipment and vehicles that are approaching pedestrians, allowing them to respond effectively by evacuating or pausing work. In addition, as shown in the subjective workload evaluation results, it has the advantage of not hindering work efficiency and concentration while improving the safety of the pedestrians.

Nevertheless, the smart glasses-based personal PWS has a few drawbacks. Smart glasses can cause discomfort to the wearer when they are worn over regular glasses or if they slip. Moreover, it would be difficult to don these smart glasses over industrial goggles and soundproof headsets, which are typically worn by workers for visual and hearing protection in mining and construction sites. Furthermore, workers may find it difficult to manipulate the smart glasses via the touchpad controller during work. To supplement this problem, the smart glasses should be controlled by motions, gestures, and voice recognition using a camera, microphone, and multi-axis sensors.

### 4.3. Comparision of Smart Glasses-Based Personal PWS with the Existing System

To compare the operating performances of the smart glasses-based personal PWS and the existing PWS, the recall for the warning alerts was estimated by investigating the number of warning alerts based on alert type. The smartphone-based PWS, which was developed by Baek and Choi [23], was used in the comparison of operating performance. The field experiment results of two PWSs were used to estimate the recall for the warning alerts. The conditions of this experiment were identical to those for the Bluetooth beacon model (RECO), i.e., the signal transmission strength was 4 dBm and the speed of approaching equipment was 10 m/s. The warning zone and the warning buffer zone were set in the ranges of 0–10 m and 10–20 m between the pedestrian and the equipment, respectively. The facing angles between the pedestrian and the equipment were fixed at 0° during the 50 trials of the existing PWS and were set at 45° intervals from 0° to 315° during the 40 trials of the proposed personal PWS. The type of warning alert and the recall are defined as follows:

- True positive: The alert was activated before the equipment approached the warning zone.
- False negative: The alert was not activated even after the equipment entered the warning zone.
- Recall: Ratio of true positives to the sum of true positives and false negatives.

Table 5 lists the recall for the warning alerts of the two PWSs. In both PWSs, true positive alarms were issued for all cases, and false negatives were not observed. Moreover, both PWSs had a recall of 100%. Thus, there was no difference in the performance of the false alarms in alert activation. Therefore, it is expected that the smart glasses-based personal PWS can replace existing PWSs and also can be employed to ensure personal safety in construction and mining sites. 

## 5. Conclusions

In this study, we developed a personal PWS that uses smart glasses to receive BLE signals from Bluetooth beacons and to provide visual proximity alerts to pedestrians. The performance assessment of the personal PWS at the mining site confirmed that the application provided a visual proximity alert along with a picture of the equipment that was approaching the pedestrian. The average BLE signal recognition distance of the smart glasses was about 37.4 m when the excavator approached from the front of the worker and about 19.4 m when the excavator approached from the rear side of the worker. The workload for the personal PWS on 10 subjects was quantitatively analyzed using the NASA-TLX criteria, which demonstrated that using smart glasses to provide visual proximity alerts led to lower mental efforts, and freed the worker’s hands, thus maintaining work efficiency. The overall workload score calculated when using smart glasses was lower than that when using a smartphone-based PWS, suggesting that smart glasses are suitable as devices for implementing personal PWSs in construction and mining sites.

Personal PWSs using smart glasses have the following remarkable advantages in construction and mining sites. First, since smart glasses are worn on the face, workers can use both hands freely. Second, pedestrians can be provided directly with visual proximity alerts, allowing them to quickly determine dangerous situations and quickly evacuate. Finally, the proposed personal PWSs could be implemented and utilized at mining and construction sites by distributing multiple sets of smart glasses and Bluetooth beacons to working sites, regardless of the scale of these working sites.

In future work, the smart glasses-based personal PWS can be extended by developing new functions through Android application programming interface. For example, Wi-Fi communication enables data exchange with remote offices, and GPS sensors can easily recognize the worker’s location. The application also recognizes the worker’s motion as the glasses contain accelerometers, gyroscopes, and geomagnetic sensors, and they can identify gestures using a camera, making it easier for pedestrians to control the smart glasses at construction and mining sites.

## Figures and Tables

**Table 1:** Specifications of commercially available smart glasses [39].

Properties	Products	
	Google Glass Enterprise Edition 2 (Google, Menlo Park, CA, USA)	Microsoft HoloLens 2 (Microsoft Corporation, Redmond, WA, USA)
**Processors**	Qualcomm Quad Core (Qualcomm, San Diego, CA, USA)	Qualcomm Snapdragon 850 (Qualcomm, San Diego, CA, USA)
**Operating system**	Android 8.0 Oreo (Google, Menlo Park, CA, USA)	Window 10 (Microsoft Corporation, Redmond, WA, USA)
**RAM**	3 GB	2 GB
**Field of view**	Diagonal 80°	Diagonal 52°

**Table 2:** Specifications of RECO beacon used for development of personal PWS.

Model		RECO Beacon
Dimensions (Diameter × Height)		45 mm × 20 mm
Weight		11.6 g (0.4 oz)
Processor		32-bit ARM^®^ Cortex^®^-M0 (ARM Holdings, Cambridge, UK)
Chipset		Nordic nrf51822 (Nordic Semiconductor, Oslo, Norway)
Casing		Acrylonitrile Butadiene Styrene (ABS) Plastic
Battery		CR2450 Lithium Coin Battery (Panasonic, Osaka, Japan)
Operating Temperature		−10 ~ 60 °C (14 ~ 140 °F)
Signal transmission period		Min (100 ms), Max (2 s)
Tx-power		Min (−16 dBm), Max (4 dBm)
Signal range		1 ~ 70 m (Directional), 1 ~ 30m (Omni-directional)
Certification	South Korea	Korea Certification (KC)
	USA	Federal Communication Commission (FCC)
	Europe	Conformité Européene (CE) marking
	Japan	Ministry of Internal Affairs and Communications (MIC) of Japan

**Table 3:** Specifications of Moverio BT-350 smart glasses (Epson, Suwa, Nagano, Japan).

Model	Moverio BT-350
Size (D × W × H)	193.5 × 176 × 30 mm (Headset), 116 × 56 × 23 mm (Controller)
Weight	119 g (Headset), 129 g (Controller)
Display device type	Si-OLED (Silicon-Organic Light-Emitting Diode)
Display size	0.43 inch wide panel (16:9)
Pixel number	921,600 pixels (1280 × 720) × RGB (3)
Field of view (FOV)	Approx. 23°
Processor	Intel® Atom™ x5 1.44GHz quad-core (Intel, Santa Clara, CA, UAS)
Operating System	Android 5.1 (Google, Menlo Park, CA, USA)
Internal memory	2 GB RAM
Camera	5 million pixels
Sensors	GPS/Gyroscopic/Accelerometer/Geomagnetic/Ambient light
Connectivity	Wi-Fi 802.11a/b/g/n/ac Bluetooth 4.1 (Bluetooth Smart Ready class 2)

**Table 4:** Results of statistical analysis of the BLE signal recognition distance (m) of the smart glass according to facing angles between the pedestrian worker and the excavator.

BLE Signal Recognition Distance (m)	Facing Angle between the Pedestrian Worker and the Excavator							
	0°	45°	90°	135°	180°	225°	270°	315°
Mean	37.4	37	35.2	15	19.4	16.8	28.2	35.2
STD ^1^	1.02	1.26	4.96	1.10	1.02	4.07	2.79	2.64
Max ^2^	39	39	40	16	21	22	31	40
Min ^3^	36	36	26	13	18	10	23	32

^1^ Standard deviation, ^2^ Maximum value, ^3^ Minimum value.

**Table 5:** Comparison of warning alert recall between smart phone-based PWS [23] and smart glasses-based personal PWS (from this study).

Type of Warning Alert	Type of PWS	
	Smartphone-Based PWS	Smart Glasses-Based Personal PWS
**Number of trials**	50	40
**Number of true positives**	50	40
**Number of false negatives**	0	0
**Recall (%)**	100	100
